# The evaluation of feeding, mortality and oviposition of poultry red mite ( *Dermanyssus gallinae*) on aging hens using a high welfare on-hen feeding device

**DOI:** 10.12688/f1000research.26398.1

**Published:** 2020-10-22

**Authors:** Francesca Nunn, Kathryn Bartley, Javier Palarea-Albaladejo, Alasdair J. Nisbet

**Affiliations:** 1 Vaccines, Moredun Research Institute, Edinburgh, EH26 0PZ, UK; 2 BIOSS, Biomathematics & Statistics Scotland, Edinburgh, EH9 3FD, UK

**Keywords:** poultry red mite, feeding device, high welfare

## Abstract

A study was performed to examine any effect of hen age on the feeding ability and mortality of different life-stages of *Dermanyssus gallinae* [Poultry Red Mite (PRM)] when fed using a high welfare, on-hen mite feeding device. Mite feeding assays were carried out every two weeks on a cohort of five Lohman Brown hens with devices containing adult and deutonymph PRM or adult and protonymph PRM. Feeding rates and mortality of each PRM life stage and oviposition of adult female PRM were evaluated over an 18-week period. There was a significant reduction in oviposition rates of female PRM as they fed on hens of increasing age. However, no clear trend was detected between the feeding rates of all three haematophagous life stages and hen age. The same conclusion was reached regarding mite mortality post-feeding in both deutonymph and adult female PRMs, although a weak positive association was apparent between hen age and protonymph PRM mortality. This study shows that the on-hen feeding device can be used both for short term studies to assess novel anti-PRM products (new acaricides, vaccines etc.) and longer, longitudinal studies to determine longevity of the effects of such novel anti-PRM products. It also demonstrates that blood feeding by mites on older hens is less able to sustain PRM populations than feeding on younger hens. This on-hen mite feeding device directly impacts upon reduction and refinement by greatly reducing the numbers of birds required per experimental group compared to traditional PRM challenge infestation models and by eliminating the need for birds to be exposed to large numbers of mites for extended periods of time that can cause welfare concerns. This paper describes the methodology for these studies and how to assemble pouches and handle mites both before and after feeding assays.

Research highlights **Scientific benefits:**

- Enables small-scale longitudinal trials to test novel mite control compounds and interventions.
- The on-hen feeding device can be used on hens of any age up to 36 weeks without affecting feeding rates or baseline mortality of all blood feeding life stages of *Dermanyssus gallinae.*
- Allows for more reliable testing of the efficacy of novel mite control compounds.

**3Rs benefits:**

- Reduction: Only requires small experimental groups of hens. This greatly reduces the number of field trials needed, which typically require several hundred hens per group in randomised block design trials.
- Refinement: *In vitro* testing demands invasive sampling of hens and unreliable *in vitro* tests require high numbers of replicates, requiring even more invasive sampling. The on-hen feeding device requires no invasive sampling.
- Refinement: The use of these devices allows the birds to remain free from the parasites for the vast majority of the experimental period, with parasites only accessing the birds for short (three hour) periods every 2-3 weeks instead of the continual exposure encountered in field trials.
- Hens can be housed at a low stocking density in high quality and environmentally-enriched floor pens, which is supportive of natural behaviours, whereas field trials necessitate the usage of commercial style cage systems where hens are kept at high stocking density in minimally enriched environments.

**Practical benefits:**

- Materials are not specialised or expensive and all are readily commercially available.
- Enables small scale, robust testing of novel control compounds before the expense and complexity of a field trial.
- Does not require housing for large numbers of hens.
- Mite feeding assays are easily carried out within a working day.

**Current applications:**

- For use in testing of vaccines for efficacy in short and in longitudinal studies against poultry red mite.

**Potential applications:**

- For use in testing novel systemic acaricides, feed additives or water additives for efficacy in short and in longitudinal studies against poultry red mite.
- Use in other blood feeding parasite-host models.
- Could be employed in disease transmission studies.

## Introduction

**Figure 1.**
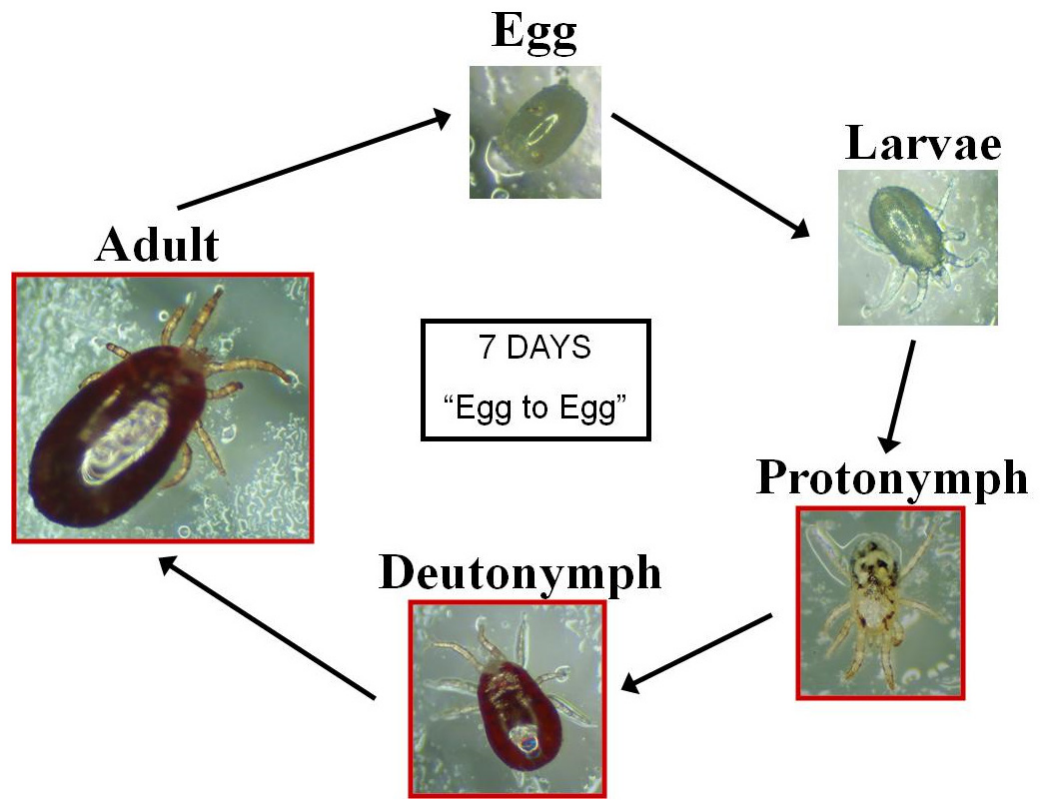
Life cycle of poultry red mites. Eggs hatch into six-legged larvae which moult into octopod protonymphs. Deutonymphs are markedly smaller than the adult females. Both protonymphs and deutonymphs must feed in order to moult to the next stage. In optimal conditions, it can take just seven days to complete its reproductive cycle. Image K. Bartley, used with permission.

Poultry red mite ( *Dermanyssus gallinae* De Geer, 1778) costs the hen egg-laying industry in excess of €231 million per year in the European Union ( Flochlay *et al.*, 2017) and is a major welfare problem in the egg laying industry where the birds are kept *in situ* for long periods of time (~1 year) allowing them to form large populations in the hens’ accommodation. This blood-feeding ectoparasite has a five-stage life cycle ( Figure 1) and the protonymph, deutonymph and adult stages are all haematophagous, feeding on the hens at night time. Even moderate infestations of mites (approximately 50,000 parasites per hen), have a negative impact on hen welfare, leading to behaviours such as restlessness at night, feather pecking and cannibalism. Severe infestations (500,000 mites per hen) can induce substantial welfare issues including anaemia, death and losses in production ( Sparagano *et al.*, 2014; Van Emous, 2005). Not only do the infestations have direct effects on hen welfare and production, *D. gallinae* is also recognised as a vector for several key avian and zoonotic diseases. These include *Salmonella* Enteritidis ( Sparagano *et al.*, 2014) and avian influenza virus ( Sommer *et al.*, 2016) and PRM has been demonstrated to act as a reservoir for fowl typhoid between sequential hen flocks ( Pugliese *et al.*, 2019).

 Many of the current chemotherapeutic control methods are ineffective, leading to unsuppressed infestations with the associated severe welfare issues and commercial losses. This had led to a recent surge in scientific research activity for novel control and treatment methods ( Flochlay *et al.*, 2017; Sparagano *et al.*, 2014).

Testing potential new mite treatments generally uses a small numbers of mites in *in vitro* efficacy assays. Positive results are followed by field testing, which by necessity involves large numbers of parasites and hens in order to account for the many variables involved in infestation of hens within a henhouse (e.g. Bartley *et al.*, 2015; Bartley *et al.*, 2017; Thomas *et al.*, 2018; Wright *et al.*, 2016). The *in vitro* method of testing novel systemic control compounds (e.g. novel vaccines, systemic acaricides in feed and water), while convenient, has in the past suffered from a high background mite mortality, device failure and variable feeding rates ( Bartley *et al.*, 2015; Wright *et al.*, 2016). These shortcomings mean that a high replicate number is required, thus leading to increased invasive sampling of hens. Bartley *et al.* (2017) demonstrated that results obtained using the *in vitro* device for testing vaccine efficacy do not always lead to mite population reduction in a field trial situation.

The experimental design of previous field trials incorporated a large number of hens (n = 768) arranged in a cage system (e.g. Bartley *et al.*, 2017) with substantial replicate blocking of the test and control groups to account for potential spatial variation in mite population growth that can occur due environmental differences across the poultry accommodation ( Moro *et al.*, 2009). These trials therefore involve large numbers of birds continually exposed to parasites for prolonged periods. In addition to the impacts of poultry red mites on hen health and welfare described for prolonged mite exposure ( Van Emous *et al.*, 2005; Sparagano *et al.*, 2014), a rise in corticosterone and adrenalin levels in continually-infested hens is indicative of moderate to severe levels of stress ( Kowalski & Sokół, 2009).

To address these issues, we have developed a novel on-hen, *in vivo* feeding device for mites and we previously described the optimisation and development of this methodology for all hematophagous life stages of the parasite ( Nunn *et al.*, 2019). This method offers an alternative to the *in vitro* tests, to allow much more accurate and reliable assessment of vaccines and other mite control methods on small numbers of hens before progressing to field studies. This strategy addresses the "Reduction" aspect of the 3Rs principles by greatly reducing the number of hens used, as it would accurately discriminate between effective and poorly-performing vaccines or other control methods (e.g. new pesticides) before they were progressed to field trials. We propose that this on-hen system may also be used to test the effectiveness of mite control methods across prolonged periods on small numbers of hens (<10 per treatment group, as opposed to several hundred per treatment group previously used in field trials ( Bartley *et al.*, 2017; George *et al.*, 2010) without continually exposing birds to the parasites, and the ability to use the device in such circumstances is the subject of this current study. This would address a second 3Rs principle - "Refinement" as it would allow the birds to remain free from the parasites for the vast majority of the experimental period, with parasites only accessing the birds for short (three hour) periods every 2–3 weeks instead of the continual exposure encountered in field trials.

There is substantial potential for using such a system in the development of novel interventions for PRM: Specifically, for vaccine testing, there are currently at least four active academic teams worldwide involved in early-stage vaccine development. Once tested in the laboratory, these vaccines will require field testing over prolonged periods to test duration of immunity and correlation with protection. If each group produces three different vaccines and, if the optimised on-hen *in vivo* feeding device was able to detect poor duration of vaccine efficacy in two of the methods for each group, that would lead to an overall reduction in hen use of ~3,000 hens in field trials. One of these groups has recently started an antigen-identification programme ( Makert *et al.*, 2016) and has identified a group of 10 proteins of interest as vaccine antigens through a combined immunoproteomics/ *in vitro* feeding device method. One of the proteins that this group has identified is identical to one on the list of 22 that we previously published ( Wright *et al.*, 2016) but the other nine are unique. If each of these proteins were to be used in field trials, this would use ~7000 birds (based on previous field trial design parameters) whereas pre-screening each of the nine candidates with an optimised on-hen *in vivo* feeding device would use ~100 birds and could therefore substantially reduce the numbers of hens entering subsequent field trials.

Beyond vaccine development, several academic groups worldwide are involved in the development of novel acaricides for PRM, using in-feed and in-water applications as well as sprays. These include the use of essential oils, combinations of essential oils and entomopathogenic fungi and novel botanicals as well as groups investigating novel uses for existing acaricides including macrocyclic lactones, amitraz, carbamates and synthetic pyrethroids. In addition to these University and Research institute-based academic teams, multiple Contract Research Organisations (CROs) are involved in testing new compounds against PRM. Each of these academic and industry applications could exploit the technology being developed in the work described herein to refine and select the most appropriate candidates for field trials before performing large-scale trials with products which may not behave in the same way on-hen as they do *in vitro*.

The purpose of the study described here is therefore to evaluate feeding and mortality of all blood-feeding life stages, and oviposition rates of adult female PRM on aging hens to assess the device’s utility for longer-term longitudinal trials.

It is envisaged that benefits arising from this research will be cross-disciplinary, benefiting researchers working on methods of controlling ectoparasites of livestock and humans as well as those working on vector-borne disease. The successful development of a method for assessing novel interventions for parasites and disease vectors before large-scale field trials are performed will promote the uptake of the 3Rs principles by greatly reducing the number of birds required per study, and is a refinement compared to the traditional infestation model that requires birds to be exposed to large numbers of mites for extended periods of time. It will have the added economic benefit of substantial reduction in the costs of product development.

## Methods

### Ethical statement

Every effort was made to emeliorate harm to the hens used in this study: the staff involved with the feeding assays were already experienced having optimised the device previously ( Nunn *et al,* 2019). Plucking of feathers was kept to a minimum, just enough to allow the pouches to be applied and plucking is not required once the hens reach ~22 weeks of age and adult feathers are present. Bandaging and tape was veterinary grade and had previously been shown to cause no harm and both handlers were practised in its usage. Hens were kept together at all times to prevent stress.

All experimental procedures described here were approved by the Moredun Research Institute Animal Welfare Ethical Review Body (approval E20/18) and were conducted under the legislation of UK Home Office Project License (reference P46F495BD) in accordance with the Animals (Scientific Procedures) Act of 1986. The manuscript was written in adherence with the ARRIVE guidelines.

### Hens

Five Lohman brown pullets (18-weeks-old at the start of the experiment), purchased from a local pullet breeding facility, were housed in an enclosed, loose litter floor pen of 2.5 m × 2 m with temperature (18 °C) and lighting (16 h light/8 h dark) controlled to mimic commercial hen laying conditions. Hens were in good condition with no evidence of previous exposure to *D. gallinae.* Hens had *ad libitum* access to layer’s pellets and water, perches and nest boxes were provided.

### Parasite material

For provision of parasite material, mixed stage and sex *D. gallinae* were collected every two weeks into vented 75 cm ^2^ canted tissue culture flasks (Corning, New York, USA) from a commercial egg laying unit. The optimum quantity of collected mites/detritus placed into a tissue culture flask was ~ 2-3 cm deep when the flasks are upright. Less than this quantity and mites desiccated over the subsequent “conditioning period”; more than this and mites were overcrowded and died. Any overcrowded culture flask (becomes wet and dirty inside) had the contents transferred into fresh flask(s) as many times as required during the seven days after collection, when the flasks were stored at room temperature (RT). Adult females and deutonymphs were isolated (see Figure 1) from this ‘mite pool’ for feeding assays following conditioning.

Protonymphs were provided by hatching mite eggs and allowing the emerging larvae to moult, as follows: Mite eggs were harvested by collecting freshly fed mites from the caps of 75 cm ^2^ tissue culture flasks and transferring ~1 cm ^3^ fed mites to individual 30 ml polystyrene Universal tubes (Thermo Scientific, Mass. USA), sealed with a double layer of AeraSeal™ film (Sigma, MO, USA) and incubated overnight at 25°C and 85% relative humidity (RH). The tubes were unsealed and placed onto glass Petri dishes in a containment moat of Flash™ multipurpose cleaning fluid (Procter and Gamble, Harrogate, UK) in a 250 ml plastic weighing boat (Scientific Laboratory Supplies, Nottingham UK). Mite eggs were then able to be picked out of the polystyrene Universal tube using a fine paintbrush and collected in 5 ml polystyrene bijoux tubes (Thermo Scientific, UK), sealed with a double layer of AeraSeal™ film (Sigma) and incubated at 25°C and 85% RH for a three-day period to allow eggs to hatch into hexapod larvae and these larvae to then moult into octopod protonymphs. The protonymphs were stored in the bijoux tubes and stored at 4°C until use. This protonymph hatching protocol made counting and handling of the small protonymphs for subsequent use easier than attempting to pick them from the mites and detritus from ‘mite pool’ field collections.

For mite conditioning prior to feeding assays, deutonymphs and adults were stored in detritus in vented 75 cm ^2^ canted tissue culture flasks (Corning) at RT for seven days after field collection to allow for complete digestion of the blood meal. All stages of mites were then stored at 4°C for two weeks (deutonymphs) or four weeks (protonymphs and adult females) before being used in feeding assays. These optimal conditioning periods had previously been empirically determined to be necessary for maximum feeding of each stage ( Nunn *et al.*, 2019).

### Experimental design and statistical analysis

Assays were carried out, on the same hens as they aged, at two-week intervals over an 18-week period. At each assay point hens either wore feeding devices containing 50 adult female mites and 50 protonymph mites or 50 adult female mites and 50 deutonymph mites. Hens were randomly-allocated feeding devices (“haphazard allocation”) and assays were carried out on Tuesday and Thursday mornings in each week in which assays were performed. At the end of the feeding period, hens were selected randomly (“haphazard selection”) for removal of the feeding devices. Blinding of hen identification numbers to the mite counter was considered to be unnecessary during experimental procedures and analysis as the single experimental group had no specific treatments to be compared with another group. Preliminary work carried out during the optimisation of the feeding device, had demonstrated that the nymph stages of *D. gallinae* feed better when in the presence of adult mites (“co-operative” feeding; Table 1).

**Table 1a.** Raw data from earlier work on the effects of including adult female poultry red mites with nymph stages on nymph feeding, when the two stages were co-fed on hens in on-hen feeding devices. Devices were left on the hen for three hours in all experiments. Table 1a shows data from two experiments with feeding devices in which no adults were included and Table 1b shows data when adults were included in the feeding devices containing nymphs. These data were taken from studies during optimisation of the on-hen feeding device. As feeding the different lifestages simultaneously is more convenient as well as improving feeding data, this was done in all subsequent trials.

Hen No.	Nymph type	Exp. 1. Fed (3h)	Unfed (3h)	Exp. 2 Fed (3h)	Unfed (3h)
1	protonymph	1	58	1	41
2	protonymph	0	57	0	42
3	protonymph	0	45	0	38
4	protonymph	7	42	0	44
5	deutonymph	5	36	0	56
6	deutonymph	0	37	4	15
7	deutonymph	3	43	0	26
8	deutonymph	1	54	0	43

**Table 1b.** Raw data from earlier work on the effects of including adult female poultry red mites with nymph stages on nymph feeding, when the two stages were co-fed on hens in on-hen feeding devices. Devices were left on the hen for three hours in all experiments. Table 1a shows data from two experiments with feeding devices in which no adults were included and Table 1b shows data when adults were included in the feeding devices containing nymphs. These data were taken from studies during optimisation of the on-hen feeding device. As feeding the different lifestages simultaneously is more convenient as well as improving feeding data, this was done in all subsequent trials.

Hen No.	Exp. 3 Adults fed (3h)	Adults unfed (3h)	Nymph type	Nymphs fed (3h)	Nymphs unfed (3h)	Exp. 4 Adults fed (3h)	Adults unfed (3h)	Nymphs fed (3h)	Nymphs unfed (3h)
1	8	2	Protonymph	0	49	4	5	3	41
2	4	6	Protonymph	15	36	10	8	7	50
3	7	5	Protonymph	7	51	5	7	2	37
4	4	2	Protonymph	6	44	4	8	7	41
5	6	3	Deutonymph	5	41	11	0	11	41
6	5	2	Deutonymph	4	13	9	2	10	30
7	7	8	Deutonymph	4	45	5	5	7	36
8	7	3	Deutonymph	17	30	5	5	11	31

Power calculations were based on simulated mite feeding data using a binomial generalised linear mixed model (GLMM) to establish the numbers of hens required to detect a change from expected scenario of 40% to worst case scenario of 15% in mean feeding rate, with baseline values for variability in feeding rate within bird over time (SD = 0.25) and between birds (SD = 0.6) estimated from two pilot previous assays using birds of different ages ( Table 2). The estimated time SD was multiplied by two to consider the case of feeding rates doubling in variability over an extended trial. The results indicated that four birds is the minimum to reach the standard 80% statistical power threshold at a 5% significance level under those conditions, however we used five to cover for unexpected deaths in this long study.

**Table 2.** Raw data from pilot studies carried out during feeding device optimisation, used to inform statistical model. Three groups of four hens had the on-hen feeding device in place for three hours. Each device contained around 50 adult female mites and Assay 1 was carried out when hens were aged 18 weeks and Assay 2 when hens were aged 22 weeks. Groups 1 and 2 refer to “Control” groups of hens in vaccine trials.

Assay 1. Hen identification no.	Group	Fed alive	Fed squashed	unfed	Total mites
63	1	0	0	43	43
64	1	24	0	17	41
65	1	19	2	22	43
66	1	1	0	42	43
85	2	6	0	39	45
86	2	15	0	40	55
87	2	12	0	40	52
88	2	8	0	19	27
73	3	8	0	37	45
74	3	16	0	40	56
75	3	3	0	47	50
76	3	1	0	22	23
Assay 2					
63	1	39	0	34	73
64	1	24	0	28	52
65	1	9	0	47	56
66	1	21	2	37	60
85	2	12	0	31	43
86	2	22	0	39	61
87	2	7	1	30	38
88 *	2	na	na	na	na
73	3	18	0	45	63
74	3	5	0	42	47
75	3	12	1	50	63
76	3	13	1	38	52

*Feeding device detached;-no feeding.

To compare mite mortality and feeding rates over time, GLMMs were fitted by the maximum likelihood method, using the Laplace approximation and a logit link function, to the data including lifestage (protonymph, deutonymph or adult), time (weeks after the start of the experiment) and the interaction between them as fixed effects and hen identity as a random effect. No criteria were set a priori for inclusion/exclusion of animals or data points although one data point was excluded as considered an extreme outlier in mite mortality being 21 times the upper limit of the interquartile range for the protonymph mortality data collected n that week (mortality 57.14%, time death observed 48h, week 10). For mite mortality, time at which death post-feeding was observed was included as an additional random effect. The effect of hen aging on oviposition was formally tested using a Poisson GLMM fitted by maximum likelihood, based on the Laplace approximation and a logarithmic link function, including an offset (in logarithmic scale) to account for the number of fed mites, with time as a fixed effect and hen identity as a random effect. Statistical testing from these model fits was conducted using type-II Wald chi-square statistics. Pairwise tests of differences in mean between lifestages and timepoints were conducted based on the predicted marginal means from the GLMM estimates. The corresponding *p*-values were adjusted for multiplicity using the Benjamini and Hochberg’s method ( Benjamini & Hochberg, 1995). Significance tests were assessed at the usual 5% significance level. All analyses were performed using R Core Team, 2018.

### Protocol for each mite feeding event

**Figure 2.**
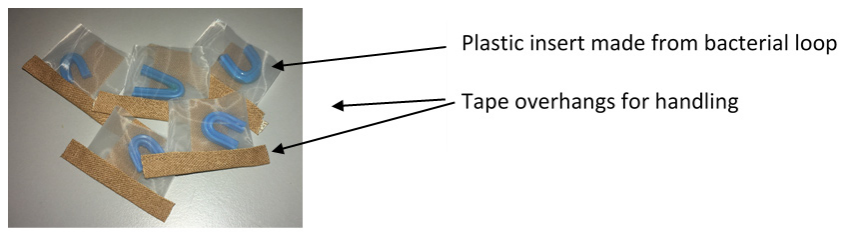
Assembled feeding devices ready to fill with poultry red mites. The tape overhangs assist with handling and the blue ‘brace’ acts both as a refuge for mites and in keeping apart the mesh sides.

***Step 1: Manufacture of feeding devices.*** Feeding devices were made in advance of each feeding event, as shown in Video 1, described in the text and shown in Figure 2.

To make the pouch ( Figure 2), a strip of polyester mesh [aperture width of 105 μm, pore depth 63 μm (Plastok Ltd, Birkenhead, UK)] was cut to 6cm wide by 15 cm long. Using 1.25 cm diameter Leukoplast® tape (BSN Medical, Germany), a seal was made on the long edge to make a tube, ensuring that the Leucoplast tape was adhered to the inside of the mesh tube. A strip of AeraSeal™ (Sigma-Aldrich Co Ltd, Dorset, UK) tape was attached to the outside of the mesh tube, to cover the Leukoplast® tape join. The tube was then cut into 3 cm segments and one of the open ends sealed with c.a. 3 cm strip of 1.25 cm diameter Leukoplast® tape, leaving an overhang at each end of the pouch. To make the plastic insert, the shaft of a blue plastic bacterial spreader loop (Quadloop Sphere, Sterilin) was gently heated in a Bunsen flame and placed onto a hard surface then bent into a U shape. The ends of the loop were trimmed and filed to remove sharp edges. The plastic inserts were then placed in pouches (one per pouch) ready to fill with mites ( Figure 2).

*Technical tips:*

1. Use fabric shears or a paper guillotine to cut the mesh to prevent the mesh from fraying.

2. Keep all edges straight to minimise the amount of sticky Leukoplast® tape adhesive that the mites come into contact with.

3. Covering the outside of the mesh tube long join with AeraSeal™ tape, covers the Leukoplast glue and makes mite recovery easier after the feeding assay.

4. Making the bottom and top seals longer than the pouch width (overhangs) enables handling of the pouches without squashing mites-it also helps in pouch removal from the hen.

Video 1 A demonstration of feeding device assembly, application of the device to hens, and device removal. Click here for additional data file. Copyright: © 2020 Nunn F et al. 2020

**Figure 3.**
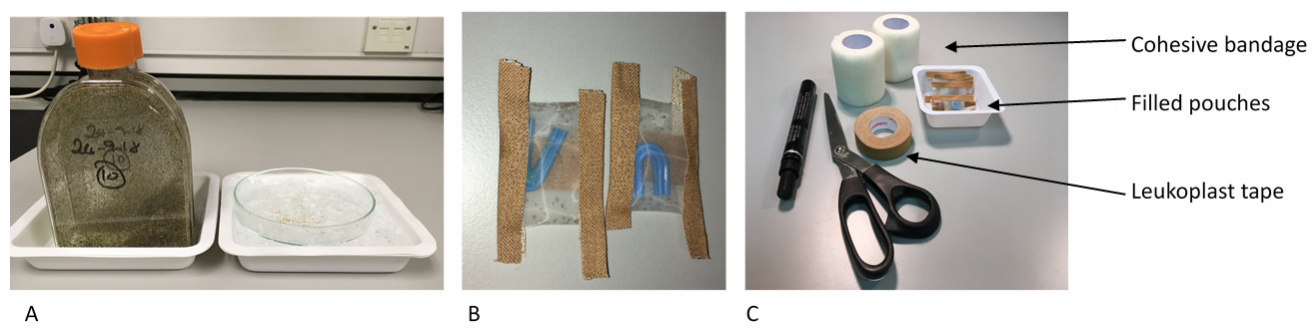
Showing poultry red mite handling using weighboats and ice (Panel A), filled pouches (Panel B) and pouch application kit (Panel C).

***Step 2: Filling the devices with mites.*** To fill the feeding devices (pouches) with mites, flasks containing conditioned mites were removed from storage at 4°C, and then kept at RT for 20–30 minutes to allow the mites to become mobile. Two centimetres of wet ice were placed into two large (280ml) chemical weigh boats (Fisherbrand). The cap of the flask was unscrewed and tapped out onto a glass Petri dish and the mites allowed to disperse for a few seconds before placing Petri dish on the ice in the weigh boat ( Figure 3a). Mites were sorted using a fine paint brush and transferred to the second glass Petri dish on ice until sufficient numbers for one feeding device were obtained. The mites are then gently gathered into a ball using the brush and placed into the feeding device which was then quickly sealed with a piece of Leukoplast® tape (BSN Medical, Germany) ( Figure 3b). Filled feeding devices were kept overnight in a loosely sealed plastic container placed in polystyrene box containing wet ice. The plastic container was raised above the wet ice on a metal support, so that it was not in direct contact with the ice. This kept the temperature sufficiently low (around 10°C) and the relative humidity level high, so that the mites did not desiccate.

*Technical tips.*

1. When transferring mites from the flask cap to Petri dish, do not use a cold Petri dish or put Petri dish on ice first as the mites will ‘ball up’ and make it very difficult to pick individuals.

2. Work quickly to limit the amount of condensation on Petri dishes - once a dish gets very wet it makes picking mites up more difficult and they won’t be in the best condition going into the feeding device.

3. When transferring the selected mites from the Petri dish to the device, place the device close to the mites on the cold Petri dish and try to transfer them in one movement. Work quickly! The blue plastic brace is a convenient place to quickly wipe the mites off the paint brush.

4. If it is convenient to fill the devices the day before application to the hens, store the devices overnight in a loosely sealed plastic container placed in an insulated box containing wet ice. Raise the container above the wet ice on a support, so that it isn’t in direct contact with the ice. This keeps the temperature sufficiently low (around 10°C) and the RH level high, so that the mites do not desiccate.

***Step 3. Application of feeding devices to hens.*** The pouch application kit is shown in Figure 3c. A video clip demonstrating pouch application can be seen in Video 1.

**Figure 4.**
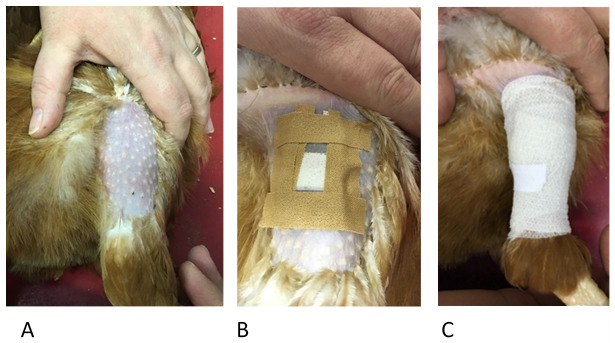
The stages of pouch application with Panel **A** showing hen restraint and plucked thigh. Hens are placed gently on their side with the handler using one hand to restrain the hen and the other to carefully straighten the leg being used. Panel **B** shows how the four strips of Leukoplast® tape are used to loosely adhere the pouch to the skin (described in Step 3) before the final bandaging using the cohesive bandage (Panel **C**). The cohesive bandage should be firm enough to be secure but not so tight as to cause any discomfort.

Prior to the first feeding assay, feathers were plucked from the outer thigh areas of each hen and thereafter whenever needed. Before laying the hen on its side, the pouch was prepared for application by cutting four strips of 1.25cm wide Leukoplast® tape. Two of those strips were cut slightly shorter for the shorter sides of the pouch. Holding the pouch with the central join facing upwards, a strip of tape was placed on each of the four sides of the pouch. The size of the pouch with the tape strips added was not larger than the plucked area of the hen’s leg ( Figure 4, panels A and B), thus preventing the tape from causing irritation to the hen while it is attached and stops the tape pulling on feathers when it is removed. The device was further secured with cohesive bandage (Central Medical Supplies Ltd., Pontefract, UK) wrapped over the feeding device which not only secures the device onto the hen but also provides dark conditions to encourage mite feeding. Feeding devices were left in place for three hours at 9am in the morning, to allow for mite processing in the afternoon. During this time hens were allowed to carry out normal foraging and nesting behaviours. Handfuls of mixed corn were scattered in the litter to encourage foraging behaviour while the pouches were attached. Following the completion of feeding (three hours after the application of the device), the devices were removed and fully engorged and partially fed mites were recovered from the devices as described below.

*Technical tips*

1. Practise bandaging on yourself or a colleague first –too tight will be uncomfortable for the hen, too loose and mites might not be able to attach for feeding.

2. A piece of leucoplast tape can be used to secure the end of the cohesive bandage if necessary.

3. If required, cohesive bandage can be cut in half (i.e. from 5 cm width to 2.5 cm width). This can be done while bandage is still on the roll, using a Stanley knife or scalpel blade.

***Step 4. Mite recovery from the feeding device.*** A video demonstrating how to dissect the pouch can be found in Video 1. The bandage can be unwound and removed completely. Slowly and carefully remove the device from the hen’s skin, peeling it off in the same direction of feather growth as this prevents damage to the hen’s skin.

**Figure 5.**
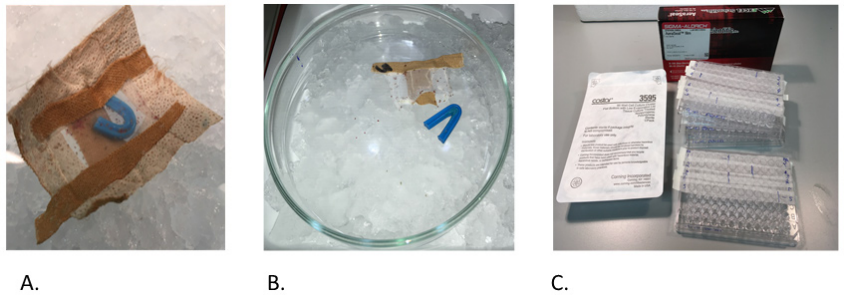
**a**) A pouch post feeding assay; **b**) partially dissected pouch; **c**) tissue culture plates containing fed mites for monitoring.

Pouches can then be dissected as shown in Figure 5a and 5b.

When taking the feeding device apart, the tape that was used to stick the pouch to the hen was removed first. Then, one of the tape seals was removed by holding one end tab on one side of the device and gently pulling the tab on the opposite side as shown (see Figure 5a and 5b). Once the seal on one side of the device had been peeled off, the blue plastic brace was then removed using forceps. The blue plastic brace and the semi-dismantled device was placed onto a glass Petri dish on ice to prevent mites escaping. Then fed/unfed mites were recovered from it. Mites generally crawled out from the feeding device during this time and were recovered from the surface of the device. The feeding device was then carefully dissected with scalpel and forceps to allow collection of and remaining mites.

Fed mites, identified by the presence of internalised hen blood, were counted, any dead mites (judged by curled legs and lack of movement) counted and then placed individually into wells ( Figure 5c) of 96-well tissue culture plates (Costar, Corning, NY, USA), sealed with AeraSeal™ tape (Sigma-Aldrich Co Ltd, Dorset, UK) and incubated at 25°C with 85% relative humidity and monitored for mortality and fecundity, 48-hour intervals for six days. Egg counting per fed mite was used as the measure for fecundity.

*Technical tips:*

1. If visualisation of the mites under light microscopy is required following feeding, isolating them in a 96-well tissue culture plates (Costar, Corning, NY, USA) allows clear microscopical visualisation of the mites through the underside of the plate. Moulting, oviposition and mortality can be scored using this system.

2. We have found AeraSeal™ to be the best tape to use as mites are less likely to stick to than to other porous tapes.

## Results

### Comparison of feeding by mite life stage and time point

**Figure 6.**
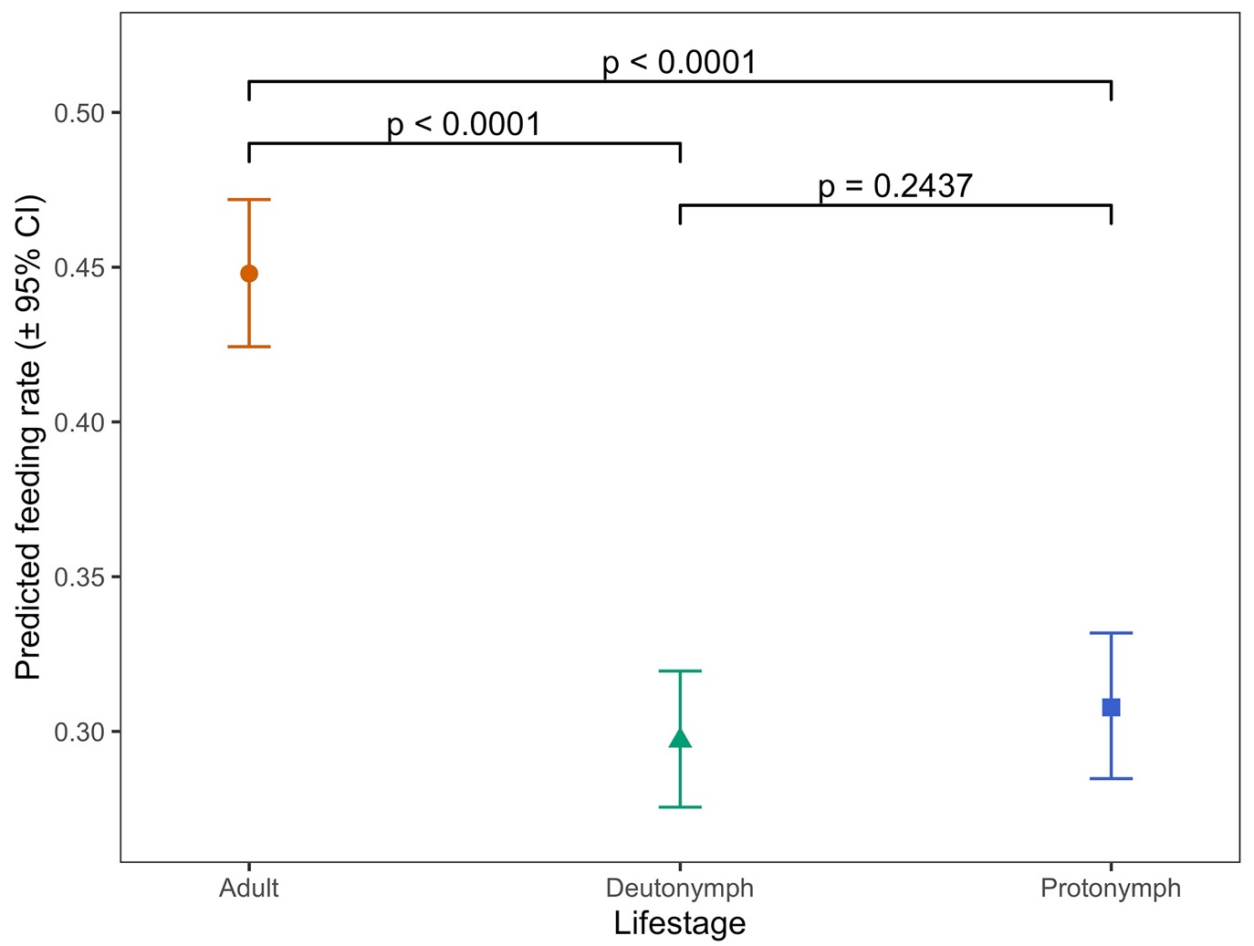
Feeding of each lifestage of Poultry Red Mites PRMs in on-hen feeding devices placed on hens across an 18-week period. Each data point represents the predicted feeding rate of mites, aggregated across all timepoints in the trial (± 95% CI) based on recorded percentages of 100 adult mites or 50 protonymph or deutonymph mites which had fed on each of five replicate hens at each timepoint.

There were statistically significant overall effects of mite lifestage (when comparing protonymph, deutonymph or adult) and of the interaction between this and time (i.e. hen aging) on the proportions of mites feeding on hens when applied to the hens in the feeding devices ( *p* < 0.0001 in each case) ( Nunn *et al.*, 2020a). The differences in predicted feeding rate (proportion of all mites in each feeding device expected to feed successfully) by mite lifestage are shown in Figure 6 (and the raw data supporting this in Table 3).

**Table 3.** Data used to generate Figure 6. Feeding rates of each lifestage of Poultry Red Mites (PRMs) in on-hen feeding devices placed on hens across an 18-week period. Each data point “rate” represents the predicted feeding rate of mites, aggregated across all timepoints in the trial (± 95% CI) based on recorded percentages of 100 adult mites or 50 protonymph or deutonymph mites which had fed on each of five replicate hens at each timepoint. “LCL” and “UCL” are the lower and upper limits of the 95% confidence interval, respectively.

Lifestage	Rate	SE	LCL	UCL
Adult	0.4480	0.0121	0.4243	0.4718
Deutonymph	0.2970	0.0112	0.2755	0.3195
Protonymph	0.3078	0.0120	0.2847	0.3318

**Figure 7.**
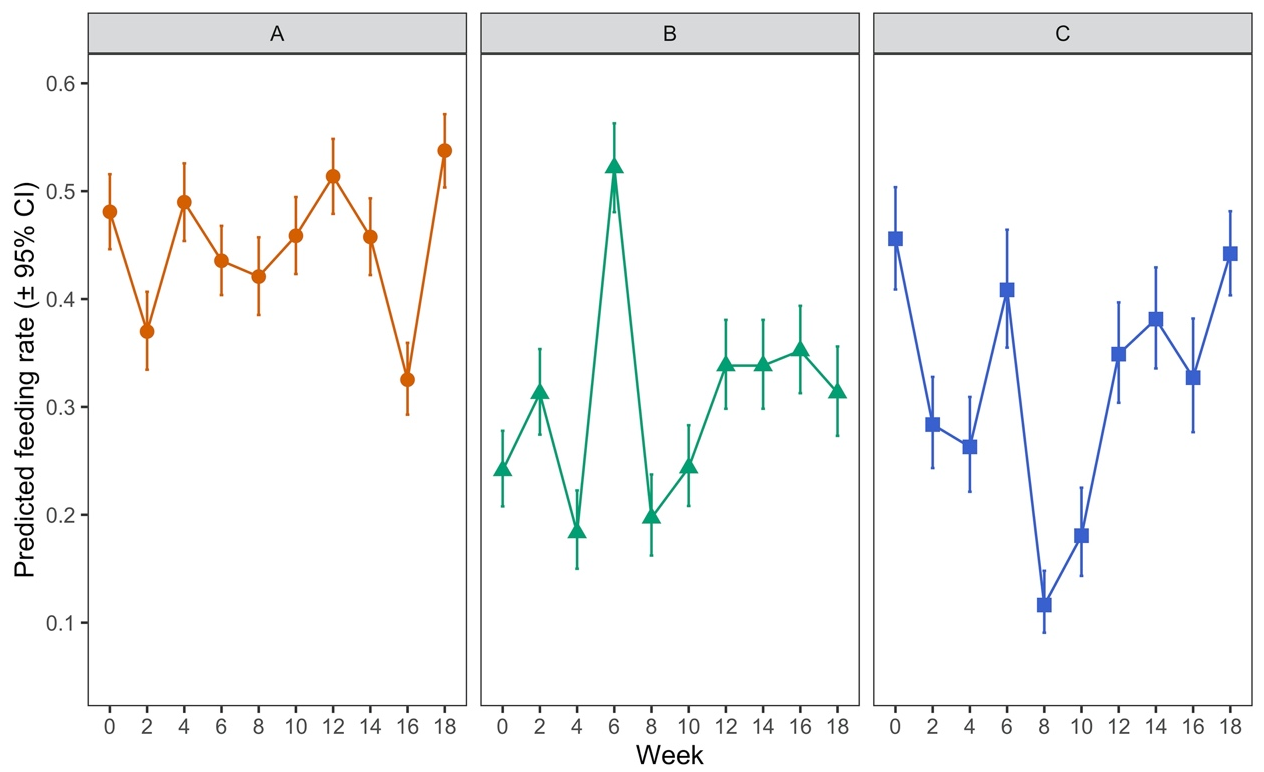
Feeding of each lifestage of Poultry Red Mite (PRM) on hens across an 18-week period. Panel **A** shows data for adult females, Panel **B** for deutonymphs and Panel **C** for protonymphs. Each data point represents the predicted feeding rate of mites (± 95% CI), based on recorded percentages of 100 adult mites or 50 protonymph or deutonymph mites which had fed on each of five replicate hens at each timepoint.

Feeding rates averaged over all 10 timepoints across the 18-week period were approximately 1.5 times higher for adult mites than for either deutonymphs or protonymphs ( *p* < 0.001 in both cases) but there were no statistically significant differences between the feeding rates of the two nymphal stages ( *p* = 0.2437). When examined between timepoints in the experiment (i.e. as the hens aged) there was notable variation in feeding rates at different timepoints for deutonymphs and protonymphs ( Figure 7, panels B and C with supporting data in Table 4). For example, at week 6 deutonymphs had much higher feeding rates than at every other timepoint ( *p* < 0.0001 in each case), though no overall trend of feeding rates across time was evident ( Figure 7B). There was substantially less variation in adult feeding across timepoints ( Figure 7A), though feeding rates at week 16, for example, were significantly lower than on all other dates ( *p* < 0.05). Again, no overall trend of feeding rates across time was obvious for adult mites.

**Table 4a.** Showing raw data from Figure 7a. Feeding of each lifestage of Poultry Red Mite (PRM) on hens across an 18-week period. Table 4a shows data for adult females, Table 4b for deutonymphs and Table 4c for protonymphs. Each “rate” represents the predicted feeding rate of mites (± 95% CI), based on recorded percentages of 100 adult mites or 50 protonymph or deutonymph mites which had fed on each of five replicate hens at each timepoint. “LCL” and “UCL” are the lower and upper limits of the 95% confidence interval, respectively.

Week	Lifestage	Rate	SE	LCL	UCL
0	Adult	0.4809	0.0178	0.4462	0.5157
2	Adult	0.3699	0.0185	0.3345	0.4067
4	Adult	0.4898	0.0184	0.4539	0.5258
6	Adult	0.4355	0.0164	0.4037	0.4678
8	Adult	0.4208	0.0184	0.3852	0.4572
10	Adult	0.4587	0.0182	0.4232	0.4946
12	Adult	0.5138	0.0177	0.4790	0.5484
14	Adult	0.4576	0.0182	0.4222	0.4934
16	Adult	0.3252	0.0170	0.2928	0.3594
18	Adult	0.5376	0.0174	0.5034	0.5714

**Table 4b.** Showing raw data used to generate Figure 7b. Feeding of each lifestage of Poultry Red Mite (PRM) on hens across an 18-week period. Table 4a shows data for adult females, Table 4b for deutonymphs and Table 4c for protonymphs. Each “rate” represents the predicted feeding rate of mites (± 95% CI), based on recorded percentages of 100 adult mites or 50 protonymph or deutonymph mites which had fed on each of five replicate hens at each timepoint. “LCL” and “UCL” are the lower and upper limits of the 95% confidence interval, respectively.

Week	Lifestage	Rate	SE	LCL	UCL
0	Deutonymph	0.2411	0.0179	0.2078	0.2778
2	Deutonymph	0.3126	0.0203	0.2743	0.3536
4	Deutonymph	0.1835	0.0185	0.1500	0.2225
6	Deutonymph	0.5219	0.0211	0.4805	0.5629
8	Deutonymph	0.1971	0.0192	0.1622	0.2374
10	Deutonymph	0.2437	0.0191	0.2082	0.2830
12	Deutonymph	0.3383	0.0211	0.2983	0.3807
14	Deutonymph	0.3383	0.0211	0.2983	0.3807
16	Deutonymph	0.3522	0.0207	0.3127	0.3937
18	Deutonymph	0.3131	0.0212	0.2732	0.3560

**Table 4c.** Showing the raw data used to generate Figure 7c. Feeding of each lifestage of Poultry Red Mite (PRM) on hens across an 18-week period. Table 4a shows data for adult females, Table 4b for deutonymphs and Table 4c for protonymphs. Each “rate” represents the predicted feeding rate of mites (± 95% CI), based on recorded percentages of 100 adult mites or 50 protonymph or deutonymph mites which had fed on each of five replicate hens at each timepoint. “LCL” and “UCL” are the lower and upper limits of the 95% confidence interval, respectively.

Week	Lifestage	Rate	SE	LCL	UCL
0	Protonymph	0.4559	0.0243	0.4089	0.5037
2	Protonymph	0.2837	0.0216	0.2433	0.3278
4	Protonymph	0.2629	0.0225	0.2213	0.3092
6	Protonymph	0.4085	0.0280	0.3550	0.4643
8	Protonymph	0.1163	0.0146	0.0907	0.1481
10	Protonymph	0.1806	0.0208	0.1434	0.2251
12	Protonymph	0.3489	0.0238	0.3039	0.3969
14	Protonymph	0.3814	0.0239	0.3357	0.4293
16	Protonymph	0.3271	0.0270	0.2766	0.3819
18	Protonymph	0.4421	0.0199	0.4035	0.4814

### Comparison of mite mortality by mite life stage and timepoint

**Figure 8.**
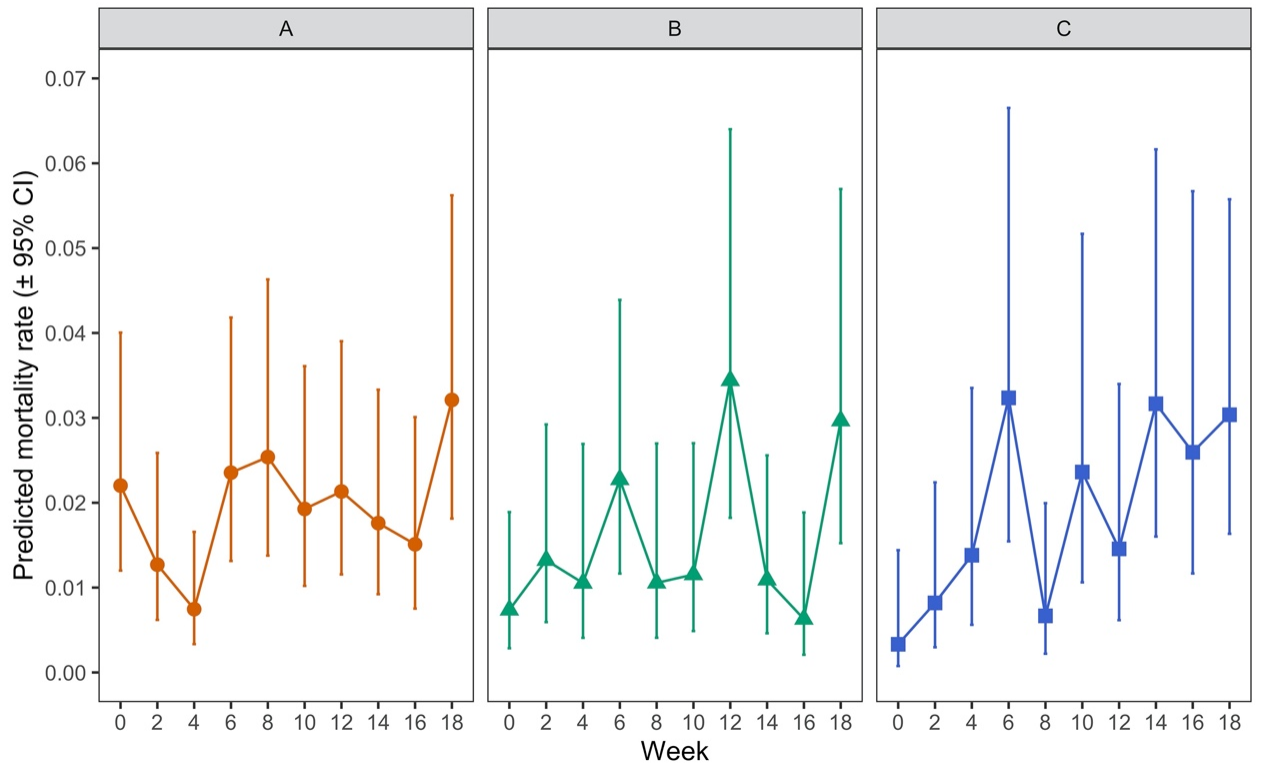
Mortality of each lifestage of Poultry Red Mite (PRM) which had fed on aging hens across an 18-week period with Panel **A** showing data for adult females, Panel **B** for deutonymphs, and Panel **C** for protonymphs. Each data point represents the predicted mortality rate of mites (± 95% CI), where a value of 1 would represent 100% mortality, based on recorded percentages of 100 adult mites or 50 protonymph or deutonymph mites which had fed on each of five replicate hens at each timepoint

There was no statistically significant overall effect of mite lifestage (when comparing protonymph, deutonymph or adult) on mite mortality post-feeding ( *p* = 0.2907). However, there was a significant effect of the interaction between lifestage and time (i.e. hen aging) ( *p* = 0.0006). This was examined further by investigating post-feeding mortality rates by time point for each lifestage ( Figure 8 and Table 5).

**Table 5a.** Data used for generating Figure 8a. Mortality of each lifestage of Poultry Red Mite (PRM) which had fed on aging hens across an 18-week period with Table 5a showing data for adult females, Table 5b for deutonymphs, and Table 5c for protonymphs. Each “rate” represents the predicted mortality rate of mites (± 95% CI), where a value of 1 would represent 100% mortality, based on recorded percentages of 100 adult mites or 50 protonymph or deutonymph mites which had fed on each of five replicate hens at each timepoint. “LCL” and “UCL” are the lower and upper limits of the 95% confidence interval, respectively.

Week	Lifestage	Rate	SE	LCL	UCL
0	Adult	0.0220	0.0068	0.0120	0.0400
2	Adult	0.0127	0.0046	0.0062	0.0259
4	Adult	0.0075	0.0030	0.0033	0.0166
6	Adult	0.0235	0.0070	0.0131	0.0418
8	Adult	0.0254	0.0079	0.0138	0.0463
10	Adult	0.0193	0.0062	0.0102	0.0361
12	Adult	0.0213	0.0066	0.0116	0.0390
14	Adult	0.0176	0.0058	0.0092	0.0333
16	Adult	0.0151	0.0053	0.0075	0.0301
18	Adult	0.0321	0.0093	0.0181	0.0562

**Table 5b.** Showing the raw data used for generating Figure 8b. Mortality of each lifestage of Poultry Red Mite (PRM) which had fed on aging hens across an 18-week period with Table 5a showing data for adult females, Table 5b for deutonymphs, and Table 5c for protonymphs. Each “rate” represents the predicted mortality rate of mites (± 95% CI), where a value of 1 would represent 100% mortality, based on recorded percentages of 100 adult mites or 50 protonymph or deutonymph mites which had fed on each of five replicate hens at each timepoint. “LCL” and “UCL” are the lower and upper limits of the 95% confidence interval, respectively.

Week	Lifestage	Rate	SE	LCL	UCL
0	Deutonymph	0.0074	0.0036	0.0029	0.0189
2	Deutonymph	0.0132	0.0054	0.0059	0.0292
4	Deutonymph	0.0105	0.0051	0.0041	0.0269
6	Deutonymph	0.0227	0.0077	0.0117	0.0439
8	Deutonymph	0.0106	0.0051	0.0041	0.0270
10	Deutonymph	0.0115	0.0050	0.0049	0.0270
12	Deutonymph	0.0344	0.0111	0.0182	0.0640
14	Deutonymph	0.0109	0.0048	0.0046	0.0256
16	Deutonymph	0.0063	0.0035	0.0021	0.0189
18	Deutonymph	0.0297	0.0100	0.0152	0.0570

**Table 5c.** Showing the raw data used for generating Figure 8c. Mortality of each lifestage of Poultry Red Mite (PRM) which had fed on aging hens across an 18-week period with Table 5a showing data for adult females, Table 5b for deutonymphs, and Table 5c for protonymphs. Each “rate” represents the predicted mortality rate of mites (± 95% CI), where a value of 1 would represent 100% mortality, based on recorded percentages of 100 adult mites or 50 protonymph or deutonymph mites which had fed on each of five replicate hens at each timepoint. “LCL” and “UCL” are the lower and upper limits of the 95% confidence interval, respectively.

Week	Lifestage	Rate	SE	LCL	UCL
0	Protonymph	0.0033	0.0025	0.0008	0.0144
2	Protonymph	0.0082	0.0042	0.0030	0.0224
4	Protonymph	0.0138	0.0063	0.0056	0.0335
6	Protonymph	0.0324	0.0121	0.0154	0.0665
8	Protonymph	0.0067	0.0037	0.0022	0.0200
10	Protonymph	0.0236	0.0096	0.0106	0.0517
12	Protonymph	0.0146	0.0064	0.0062	0.0340
14	Protonymph	0.0317	0.0109	0.0160	0.0616
16	Protonymph	0.0259	0.0105	0.0117	0.0567
18	Protonymph	0.0304	0.0095	0.0163	0.0558

Mortality rates were variable for adults and deutonymphs across the time period of the experiment. For adults ( Figure 8A), mortality at week 4 was significantly lower than at weeks 0, 6, 8 and 18 ( *p* < 0.05) and, in addition, mortality rates for adults at week 18 was significantly higher than at weeks 4 and 16 but no clear pattern over time could be discerned across the whole experiment. For deutonymphs ( Figure 8B), mortality at week 12 was significantly higher than at weeks 0, 4, 10, 14 and 16 ( *p* < 0.05) and mortality at week 18 was significantly higher than at week 16 ( *p* < 0.05). Again, no discernible trend in mortality over time could be determined for deutonymphs. In contrast, for protonymphs, there appeared to be a weak positive trend in mortality as the hens aged ( Figure 8C), with significant increases in mortality of protonymphs between week 0 and weeks 6, 10, 14, 16 and 18 ( *p* < 0.05). Overall, levels of mortality were very low in each lifestage albeit with variability in mortality rates observed for all lifestages as indicated by the wide confidence intervals.

### Comparison of mite oviposition by timepoint

**Figure 9.**
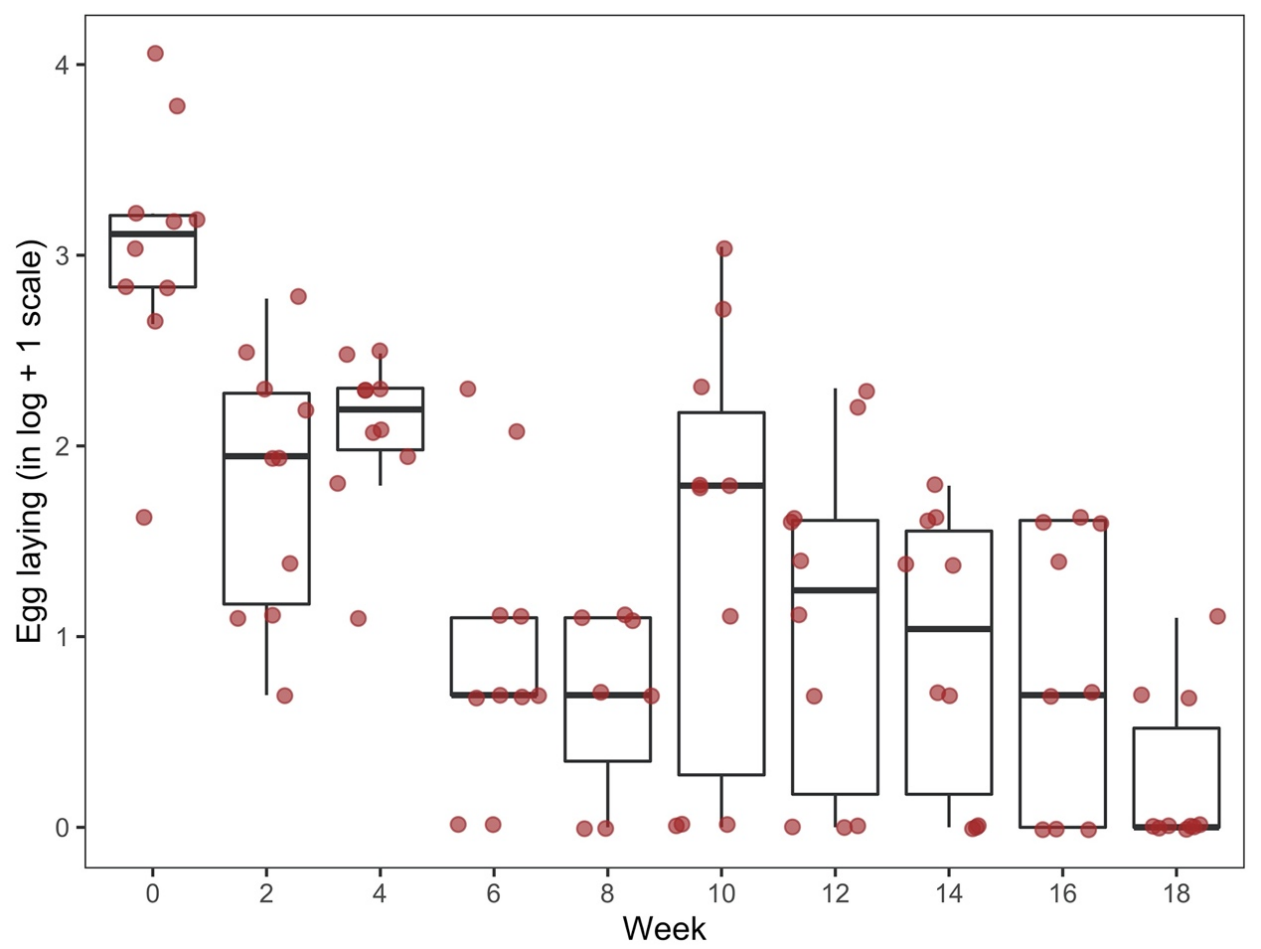
Oviposition of Poultry Red Mite (PRMs; n = 1964) which had fed on aging hens across an 18-week period (boxplots and actual values of egg counts in logarithmic scale). Data shows the total number of eggs laid per fed, surviving female mite as monitored over a six-day period following each assay week.

Analysis of the oviposition of adult mites (total eggs per surviving, fed female mite monitored over the six-day period for each time point) over time demonstrated that there was a statistically significant overall effect of hen age on the capacity of adult mites to lay eggs after feeding on these hens ( *p* < 0.0001; Figure 9 and Table 6), with a trend of oviposition dropping as the hens aged.

**Table 6.** Data used in generating Figure 9 (in log+1 scale). Oviposition of Poultry Red Mites (PRMs) which had fed on aging hens across an 18-week period (boxplots and actual values of egg counts in logarithmic scale). Data shows the total number of eggs laid per fed, surviving female mite as monitored over a six-day period following each assay week. “Q1” and “Q3” are the first and third quartiles of the data distribution, respectively.

Week	Minimum	Q1	Median	Q3	Maximum
0	1.6094	2.8332	3.1113	3.2087	4.0431
2	0.6931	1.1705	1.9459	2.2762	2.7726
4	1.0986	1.9793	2.191	2.3026	2.4849
6	0	0.6931	0.6931	1.0986	2.3026
8	0	0.3466	0.6931	1.0986	1.0986
10	0	0.2747	1.7918	2.1749	3.0445
12	0	0.1733	1.2425	1.6094	2.3026
14	0	0.1733	1.0397	1.5537	1.7918
16	0	0	0.6931	1.6094	1.6094
18	0	0	0	0.5199	1.0986

Thus, average mite oviposition at week 0 was statistically significantly higher than that at all subsequent weeks ( *p* < 0.0001) and average oviposition at week 18 was significantly lower than that at all previous timepoints ( *p* ˂ 0.0012).

## Discussion

This study demonstrated that, while feeding rates of adult females were significantly higher than those of nymphal stages, there was no evidence of a decreasing trend in feeding rates of any life stage associated with hen age, with only punctual significant deviations. Mite mortality was generally very low using this *in vivo* feeding device, certainly substantially lower than recorded when using *in vitro* feeding devices (e.g. 27% in Wright *et al.*, 2009). There were no significant overall differences in fed mite mortality rates between life stages and no significant increased mortality was observed in deutonymph or adult life stages with increased hen age, although a weak positive association was apparent in protonymphs. A significant decrease in egg-laying by fed adult females was demonstrated with increased hen age.

This effect of hen age on the egg-laying capacity of *D. gallinae* following feeding on the hens may be responsible for the observed limits in expansion of mite numbers in prolonged field trials (e.g. see Bartley *et al.*, 2017; Mul *et al.*, 2017) but the cause of the observed effect is not clear. The historical dogma is that no effective, protective, natural immune response develops in hens against PRM in natural infestations and the limited exposure (in terms of time and mite numbers) of hens to the parasite in our experiment would almost certainly preclude an effective humoral immune response. The effect may therefore come from a change in the physiology of the hens during that period making feeding less nutritionally rewarding to the mite as the hen ages.

The feeding behaviour of *D. gallinae* is poorly understood, but from our previous work it seems that nymph feeding is greatly increased when nymphs and adults are allowed to feed simultaneously ( Table 2). Further work is required to definitively assess any co-operation in adult and nymph feeding as well as between nymph stages. Feeding rates across the time course described here were variable for all life stages, although less variable for adults than for either of the nymph stages. This variability may at least in part be attributed to mite quality, which may be inconsistent as mites are collected from field populations separately for each individual feed. This could be addressed by having a continual lab supply of mites from infested hens under controlled conditions (e.g. see Wang *et al.*, 2018) or, preferably, from a continuous *in vitro* culture system. We have previously described optimisation of mite conditioning ( Nunn *et al.*, 2019) for the on-hen feeding assay and this helps in addressing some of the issues around variability in feeding rates. While a certain amount of conditioning is crucial to allow the collected mites time to digest previous blood meals, a degree of flexibility may be required in this conditioning period to maximise feeding rates ( Lima-Barbero *et al.*, 2019) and the use of real time controls for comparison is imperative in order to account for background mortality rates.

 Mite population dynamics in field infestations are not well characterised but differences in ratios of life stages have been noted previously in collections from hen houses ( George *et al.,* 2010), and have been observed in collections by our own group. The reasons for this are unclear but may relate to the variable effects of hen aging on the post-feeding survival of different life stages noted here ( Figure 3). If required for short term *in vivo* experiments to determine the effects of novel mite interventions (e.g. novel acaricides) the increase in protonymph mortality and decrease in oviposition associated with hen age, seen in this study, can be avoided in by choosing younger hens for use in mite feeding assays. Equally, for longer term, longitudinal studies, the expected effects of hen aging on the mites should be incorporated into any formal sample size calculations if the anticipated effects of the novel intervention are likely to impact protonymph mortality or adult mite fecundity.

The results of this study can therefore be used in designing shorter trials to ascertain whether a vaccine or some other systemic acaricide has any effect on fed mites. Short-term trials such as that described in Price *et al.* (2019) can be relatively easy to carry out with young hens and mites used from the same collection point to give a more consistent feeding rate. Price *et al.* (2019) demonstrated that although feeding rates were variable for three assays using the same batch of conditioned mites, there was no significant difference in feeding rates between the different treatment groups. For longer trials, designed to look at the longevity of an IgY response to novel vaccine antigens, adequate controls must be in place to assess background mortality and oviposition of the batch of the mites in use.

The exploitation of the device being developed in this proposal is not limited to the PRM - academic beneficiaries working on all ectoparasites and arthropod disease vectors including fleas, biting and chewing lice, cimicids (e.g. bedbugs) and ticks could easily adapt the device to produce a refinement of their research involving animals which would minimise parasite exposure of the test animals during blood-feeding in experiments where novel interventions (both insecticide/acaricide effects and anti-disease vector effects) are being tested and reduce numbers of animals by weeding out poorly performing interventions prior to field testing.

## Data availability

### Underlying data

Figshare: The evaluation of feeding, mortality and oviposition of poultry red mite (Dermanyssus gallinae) on aging hens using a high welfare on-hen feeding device_DATA.xlsx. https://doi.org/10.6084/m9.figshare.12848432.v1 ( Nunn *et al.*, 2020a).

Data are available under the terms of the Creative Commons Attribution 4.0 International license (CC-BY 4.0).
