# Supplementary material for: The evaluation of feeding, mortality and oviposition of poultry red mite ( Dermanyssus gallinae) on aging hens using a high welfare on-hen feeding device
Source: F1000Res. 2020 Oct 22;9:1266. [Version 1] doi: 10.12688/f1000research.26398.1 (PMC7684678; doi:10.12688/f1000research.26398.1)
Supplement: Peer review history

# Peer review: Reviewer response for version 1

**DOI:** 10.5256/f1000research.29146.r73832

**Published:** 2020-11-23

**Authors:** Antonio Camarda, Antonella Schiavone

**Affiliations:** 1 Department of Veterinary Medicine, University of Bari - Aldo Moro, Valenzano, Italy

The paper focuses on the application of an innovative on-hen *in vivo* technique for feeding mites, improving previous studies about the same topic. As explained by the authors, this device could represent an important alternative for testing new acaricides and vaccines, as well as for studying the vectorial role of *Dermanyssus gallinae*. The technique is well described and rich in details, in particular the section concerning the device construction. Furthermore, the presence of figures, videos and technical tips, which could be very helpful for both understanding and reproducing the experiment, is remarkable. Moreover, the obtained results are supported by a strong and effective statistical analysis.

 Anyway, I have few observations for the authors:

- Introduction: “If each group produces three different vaccines and, if the optimised on-hen *in vivo *feeding device was able to detect poor duration of vaccine efficacy in two of the methods for each group, that would lead to an overall reduction in hen use of ~3,000 hens in field trials.” The authors should explain how these numbers are estimated.
- Methods – Ethical statement: it is not clear the reason why plucking feathers is not necessary in hens older than 22 weeks of age. Please, clarify the concept.
- Methods – Parasite material: the paragraph sounds a bit confusing. It could be useful to adopt a short step-by-step scheme.
- Methods – Parasite material: how did you distinguish sexes and stages of mites?
- Methods – Parasite material: taking into account that the life cycle of *Dermanyssus gallinae* requires 2-3 days for eggs to hatch and 1-2 days for larvae to molt into protonymphs, can a three-day period of incubation be long enough to obtain an adequate number of protonymphs?
- Methods - Protocol for each mite feeding event – Step 1: According the other reviewer’s suggestion, 1,25 is the tape’s width and not the diameter.
- Methods – Protocol for each mite feeding event – Step 3: is there a specific reason why the device has been left in place for a three-hour period?
- Methods – Protocol for each mite feeding event – Step 4: “The feeding device was then carefully dissected with scalpel and forceps to allow collection of and remaining mites”. Did you intend any remaining mites?
- Discussion: “The reasons for this are unclear but may relate to the variable effects of hen aging on the post-feeding survival of different life stages noted here”. Could the differences in ratios of life stages observed in field collected mite populations be related, not only to the effects of hen aging, but also to the adopted mites collection technique? It could be useful to reconsider the paragraph taking into account this concept.

Is the rationale for developing the new method (or application) clearly explained?

Yes

Is the description of the method technically sound?

Yes

Are the conclusions about the method and its performance adequately supported by the findings presented in the article?

Yes

If any results are presented, are all the source data underlying the results available to ensure full reproducibility?

Yes

Are sufficient details provided to allow replication of the method development and its use by others?

Yes

Reviewer Expertise:

Dermanyssus gallinae vectorial role;  Acaricidal evaluation; Dermanyssus gallinae population dynamics

We confirm that we have read this submission and believe that we have an appropriate level of expertise to confirm that it is of an acceptable scientific standard.

# Peer review: Reviewer response for version 1

**DOI:** 10.5256/f1000research.29146.r73574

**Published:** 2020-11-10

**Authors:** Cristian Magdas

**Affiliations:** 1 Department of Parasitology and Parasitic Diseases, University of Agricultural Sciences and Veterinary Medicine, Cluj-Napoca, Romania

A very good manuscript which provide the results of a study what brings a new method for feeding of poultry red mite, very useful in testing the effectiveness of new systemic control compounds against this  mite, feeding method which could be used also for other arthropods. The manuscript brings also important data regarding the decrease in egg-laying by adult females fed on increased hen age, with the recommendation of younger hens for use in mite feeding assays. The methods are very well described, really appreciated the technical tips *.* Anyhow, I have some suggestions/questions for the authors:

- Methods-ethical statement-first line – “ameliorate”.
- Please check on the page 5 and replace “bijoux” with “bijou”. I think that the correct version is “bijou containers” and please check if not 7 ml instead of 5 ml? Also, “30 ml polystyrene Universal containers” I think is better, just a suggestion.
- 1,25 cm isn’t the width of the Leukoplast, but not the diameter?
- In the legends of figure 6, 7 and corresponding tables is mentioned “100 adult mites”. Why recorded percentages refers to 100 adult mites, but not to 50 adult mites?
- Experimental design and statistical analysis: in the week in which the assays were performed, the feeding devices were applied 2 times on the hens?
- Maybe it would be good if the authors explained why the feeding devices were left fixed for only 3 hours?

Is the rationale for developing the new method (or application) clearly explained?

Yes

Is the description of the method technically sound?

Yes

Are the conclusions about the method and its performance adequately supported by the findings presented in the article?

Yes

If any results are presented, are all the source data underlying the results available to ensure full reproducibility?

Yes

Are sufficient details provided to allow replication of the method development and its use by others?

Yes

Reviewer Expertise:

Veterinary Parasitology

I confirm that I have read this submission and believe that I have an appropriate level of expertise to confirm that it is of an acceptable scientific standard.
